# Electrochemically-Driven Insertion of Biological Nanodiscs into Solid State Membrane Pores as a Basis for “Pore-In-Pore” Membranes

**DOI:** 10.3390/nano8040237

**Published:** 2018-04-13

**Authors:** Farid Farajollahi, Axel Seidenstücker, Klara Altintoprak, Paul Walther, Paul Ziemann, Alfred Plettl, Othmar Marti, Christina Wege, Hartmut Gliemann

**Affiliations:** 1 Institute of Functional Interfaces (IFG), Karlsruhe Institute of Technology (KIT), 76344 Eggenstein-Leopoldshafen, Germany; farid.farajollahi@kit.edu; 2 Institute of Solid State Physics, University of Ulm, 89081 Ulm, Germany; dr.a_seid@gmx.de (A.S.); paul.ziemann@uni-ulm.de (P.Z.); alfred.plettl@gmail.com (A.P.); 3 Institute of Biomaterials and Biomolecular Systems, University of Stuttgart, 70569 Stuttgart, Germany; klara.altintoprak@bio.uni-stuttgart.de (K.A.); christina.wege@bio.uni-stuttgart.de (C.W.); 4 Central Facility for Electron Microscopy, University of Ulm, 89081 Ulm, Germany; paul.walther@uni-ulm.de; 5 Institute of Experimental Physics, University of Ulm, 89081 Ulm, Germany; othmar.marti@uni-ulm.de

**Keywords:** nanomembrane, nanopores, electrophoresis, self-assembly, viral nanodiscs, tobacco mosaic virus, discs, bio-inorganic hybrid material

## Abstract

Nanoporous membranes are of increasing interest for many applications, such as molecular filters, biosensors, nanofluidic logic and energy conversion devices. To meet high-quality standards, e.g., in molecular separation processes, membranes with well-defined pores in terms of pore diameter and chemical properties are required. However, the preparation of membranes with narrow pore diameter distributions is still challenging. In the work presented here, we demonstrate a strategy, a “pore-in-pore” approach, where the conical pores of a solid state membrane produced by a multi-step top-down lithography procedure are used as a template to insert precisely-formed biomolecular nanodiscs with exactly defined inner and outer diameters. These nanodiscs, which are the building blocks of tobacco mosaic virus-deduced particles, consist of coat proteins, which self-assemble under defined experimental conditions with a stabilizing short RNA. We demonstrate that the insertion of the nanodiscs can be driven either by diffusion due to a concentration gradient or by applying an electric field along the cross-section of the solid state membrane. It is found that the electrophoresis-driven insertion is significantly more effective than the insertion via the concentration gradient.

## 1. Introduction

A huge number of different top-down approaches are used to artificially modify large-scale materials by removing or by depositing material to create small structures at a nanometer scale. Generally, in lithography-based techniques, the bulk materials are modified site-selectively either in terms of their chemical or physical properties to form structures or patterns that are defined in size, shape, density, periodicity and even in the dimensionality [1]. Zhang et al. document in a recent review paper that, depending on the applied techniques, several steps of top-down techniques might be necessary to end up with desired structures such as porous membranes [2]. Porous membranes play an increasing role in many fields including ionic selective diffusion, ionic gating and ionic rectification in the solid-state nanopores and nanochannels. These nanopores and nanochannels, for their part, can be applied to molecular filters, biosensors, nanofluidic logic devices and energy conversion devices [2]. Membranes with pore diameters between 1 and 100 nm are referred to as nanoporous, as long as the pore diameter is comparable to the pore depth [3]. The available nanoporous membranes range from naturally-assembled biological cell membranes (which support ion or protein transport into or out of the cytoplasm, i.e., the inner space of a cell) [4] to artificially-produced specific membranes, e.g., those used for molecular separation in the gas phase [5] or in liquid [6]. In the last few decades, numerous methods have been developed to prepare nanoporous membranes either in the form of single pore or multiple pore membranes. For instance, ion or electron beams are proven to produce precisely-shaped single or multiple pores in different materials. As was shown by Li et al., individual pores were formed after irradiating a free-standing silicon nitride (SN) membrane with Ar^+^ ion beams [7]. These beams can be, depending on the experimental parameters, varied in diameter within the range of a few to several hundreds of nanometers [7]. Electron beams (e-beams) of transmission or scanning electron microscopes (TEM or SEM, respectively) were used as tools to drill holes into a membrane consisting of silicon on insulator wafers and free-standing SiO_2_ or SN layers [8,9,10]. Recently, the preparation of single pores in graphene membranes using e-beam lithography has been reported [11,12]. To prepare single pores in plastic materials, Wu et al. used a laser-based surface-tension-driven mass flow technique [13]. A mechanical way to produce nanopores in mica membranes has been demonstrated by using the tip of an atomic force microscope (AFM) as a machining tool [14]. All the methods mentioned above can be used to prepare individual pores, but are time-consuming and cannot be applied in large-scale high-throughput and parallel pore production. To prepare large-area multiple pore membranes, other techniques such as gold (Au) nanoparticle (NP)-assisted plasma etching [15], ion track-etching-based membrane preparation [16,17] or anodic oxidation of aluminum to obtain porous alumina and corresponding membranes have been carried out [18,19,20]. The critical drawbacks of the membrane pores prepared artificially by the top-down methods mentioned above are that their geometries are irregular in shape, and a wide pore size distribution is obtained in the case of the multiple pore membranes.

In contrast to the top-down techniques, a bottom-up approach starts with individual, but different building blocks on the molecular level. These are arranged during a self-assembly process to form bigger aggregates in a two-dimensional or three-dimensional fashion. A typical example of a three-dimensional bottom-up process is the formation of biological nanoparticles such as the tobacco mosaic virus (TMV) tubular assemblies. The TMV nanotubes consist of a single-stranded RNA molecule, helically packaged into more than 2100 coat protein (CP) units [21]. Another example of a molecules’ self-organization is the formation of artificial, highly porous and crystalline metal-organic frameworks, which are built up through the self-assembly of organic linker molecules and inorganic building blocks [22,23,24]. Besides the aforementioned methods, new materials with additional functionality can be created by a combination of both bottom-up and top-down approaches. We have recently demonstrated a site-selective deposition of viral RNA on areas prepared by a top-down dip-pen nanolithography process followed by the bottom-up self-assembly of TMV-like nanoparticles (TLNPs) [25]. Another example is the site-selective growth of surface-anchored metal-organic frameworks on a substrate that was pre-patterned by micro-contact printing or atomic force microscope (AFM) nanografting [26,27]. In other examples, the self-assembly process is carried out in a separate step, and the products are immobilized on the pre-structured substrate site-selectively. Moon et al. have reported the immobilization and alignment of *Bacillus subtilis* bacteriophage Phi29 procapsid particles in a nanoporous membrane [28]. A different strategy was used to insert a single alpha-hemolysin protein pore into the nanopore of a solid state membrane (SSM) via electrophoresis [29]. A similar approach has been used to integrate DNA origami on a glass nanocapillary under the influence of an electric field [30].

**Figure 1:**
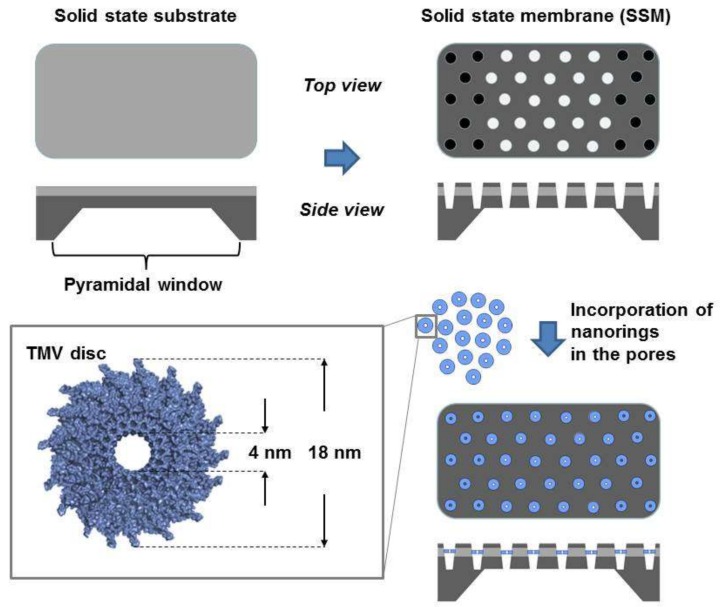
Scheme of the membrane based on the “pore-in-pore” structure. The virus-based nanorings are incorporated in the conical pores of the solid state membrane (SSM). The 4-nm pin holes of the nanodiscs may act as a molecular filter with exactly defined protein pore diameters. For the tobacco mosaic virus (TMV) disc structure, see [34,35].

In this work, we describe a combination of biological functional units with an inorganic template according to [31] by applying concentration gradient-based and electrophoresis-driven diffusion: the insertion of biologically bottom-up self-assembled TMV-based nucleoprotein nanodisc structures [32] into the hexagonally-arranged conical pores of an artificial solid state membrane (SSM, Figure 1). The SSM is prepared using a top-down micelle technique [33]. The aforementioned nanodiscs are RNA-stabilized protein assemblies, which are derived from natural building blocks of TMV particles and contain a defined number of TMV coat proteins (CPs) and an RNA of 204 nucleotides. The nanodiscs have a thickness of about 9 nm, an outer diameter of 18 nm and a central pore that has a defined diameter of 4 nm (in the case of wild type CPs). These RNA-stabilized discs are referred to as nanodiscs, nanorings or TLNPs to discriminate them from the naturally-existing, RNA-free TMV discs occurring as viral CP assembly intermediates. Due to the naturally given accuracy of the nanodiscs in terms of their diameters and regular shapes, this “pore-in-pore” preparation method can be considered a prototype protocol for the fabrication of nanofilter membranes with pores of very narrow and reproducible diameters.

## 2. Results and Discussion

### 2.1. Characterization of the Solid State Membrane and Viral Nanodiscs

**Figure 2:**
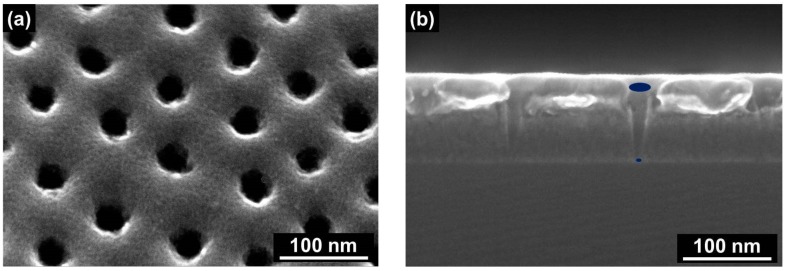
(**a**) A top view SEM image of the upper side of the SSM and (**b**) a cross-section SEM image of the conical pores of the SSM. The ellipses indicate the two different apertures of the conical pore.

The preparation method described in detail by Seidenstücker et al. [33] and briefly in the experimental part of this paper results in a conical shape of the SSM pores. This acts as a funnel, trapping the TMV nanodiscs inside the pores to avoid translocation of the nanodiscs while an electric field or a concentration gradient, respectively, conduct the charged nucleoprotein particles into the pores. The apertures of the pores on the front-side surface of the membrane are larger than the diameter of the TMV nanodiscs. This leads to an effective insertion of the nanodiscs into the pores, while the pores on the backside of the SSM have smaller diameters to keep bio-nanodiscs inside. As many parallel pores exist in the membrane, their size distribution in terms of the apertures should be in a proper range to fulfill the mentioned size requirements. Figure 2 shows the top view and the cross-section view scanning electron microscope (SEM) images of representative conical pores. 

**Figure 3:**
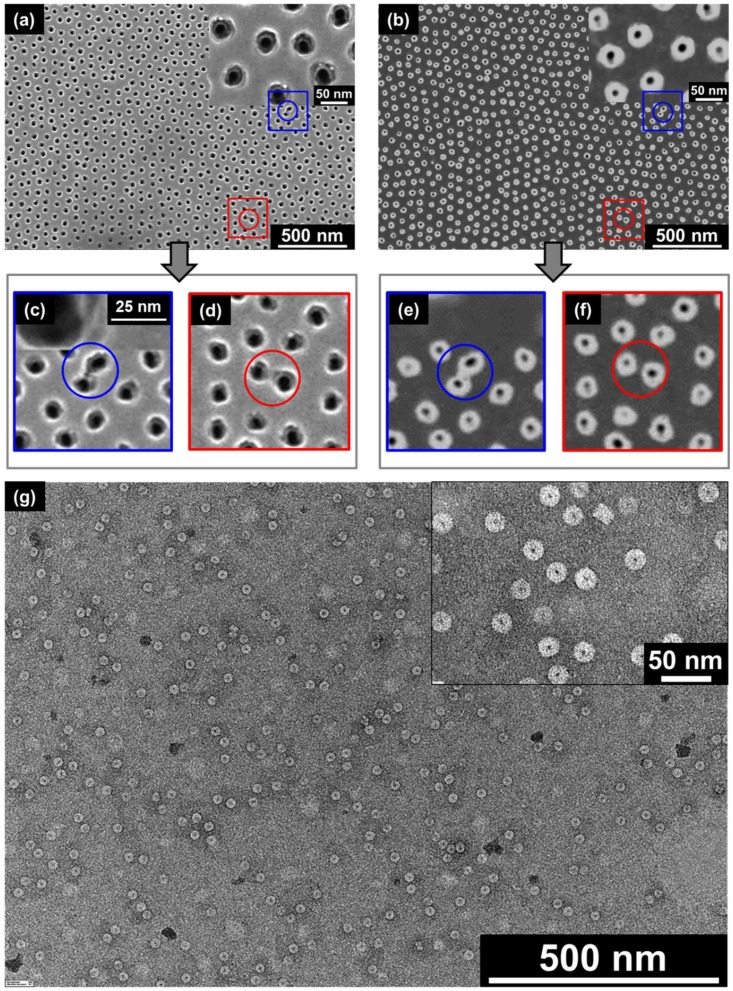
(**a**) Secondary electron top view SEM image of the SSM. (**b**) Dark field transmission mode SEM image of the backside of the SSM. (**c**,**d**) are the zoom-in images of the blue and red squares in image (**a**), respectively. (**e**,**f**) show the backside pore apertures corresponding to (**c**,**d**), respectively. (**g**) TEM image of negatively-stained TMV nanodiscs on a carbon/Formvar^®^-covered copper grid.

Using the membrane cross-section images for the statistical evaluation of the apertures of the conical pores has several drawbacks. For cross-section analysis, the membrane has to be broken and cannot be used for further experiments. In addition, it is challenging to break the membrane in a way that (1) several pores are exactly arranged along the cross-sectional area and (2) all of the pores are cut exactly along their middle axis. Therefore, the dark-field transmission mode of the SEM setup was used to differentiate between the distinct pore diameters. While Figure 3a shows an SEM image (based on secondary electrons) of a representative area at the top side of a SSM, Figure 3b shows the SEM image of the corresponding pores on the backside of this membrane area. However, here, the SEM was operated in the dark field transmission mode. While it is hard in the bright field image to differentiate between the top and the bottom pore apertures, the dark field image gives a clear contrast between them and can be used for statistical evaluation. In some cases, it seems that on the front side of the SSM, pores are interconnected (see the blue and red circles in Figure 3a). However, it is hard to differentiate between pores that are interconnected in terms of interconnected pore channels along the whole longitudinal pore axis or just in terms of overlapping inlet pore apertures. In Figure 3c,d the corresponding zoom-in images of the blue and the red square, respectively, of Figure 3a are depicted, while Figure 3e,f shows the corresponding images of the outlet pore apertures on the backside of the membrane. It can be seen that in both cases, the outlet pore apertures are clearly separated, which allows for the assumption that the pores are not interconnected along the whole length of the pore channels and can thus be seen as separated, single pores. Figure 3g presents transmission electron microscope (TEM) images of TLNPs on a flat surface. The dark areas in the middle of the nanodiscs indicate the 4-nm channels.

**Figure 4:**
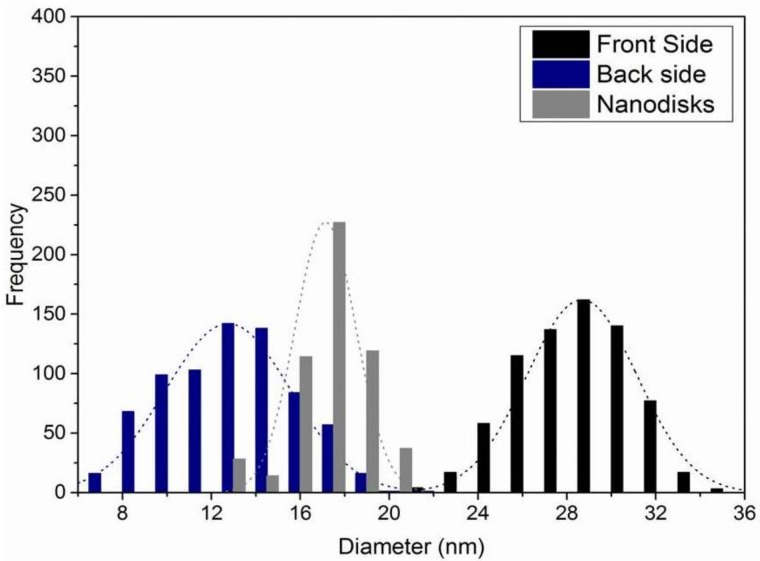
Distribution of the pore diameters for the top side (black bars) and the back side (blue bars) of the SSM. The grey bars represent the distribution of the diameters of the viral nanodiscs. A Gaussian distribution function is fitted to the three size distributions.

Based on dark field images, the size distribution of the pore apertures is calculated by ImageJ software [36] and plotted in Figure 4. For the larger pore apertures of the top side of the SSM (represented by black bars), a mean diameter of 29 nm was determined. The mean diameter of the pore openings at the back side (represented by blue bars) is 13 nm. The grey bars in Figure 4 represent the size distribution of the TMV nanodiscs. The results verify that the conical pores are appropriate traps for the nanodiscs, whose outer diameter is significantly larger than the smaller apertures of the conical pores.

### 2.2. Electrochemical Characterization of the Membrane

As described earlier, two approaches were used to incorporate the nanodiscs into the pores of the SSM. One is the concentration gradient-driven incorporation, while the second approach uses the movement of the negatively-charged nanodiscs in an electric field of an electrophoresis setup. The second approach was motivated by several works that describe that single nanopores have been frequently used to detect the translocation of small molecules, DNA, particles and viruses [37,38,39]. For all these cases, individual nanopores are usually applied so that just one single molecule or particle can pass through the pore during a very short period of time without being trapped inside the pore. During this time period, the ionic current through this pore is blocked and causes a measurable current drop between the two electrodes. In the case that a high number of pores exist in the membrane, detection of a single passage event is not possible, and current variation will reflect a statistical average of all superposed diffusion events. This leads to a significant and measurable decrease of the background current [40].

**Figure 5:**
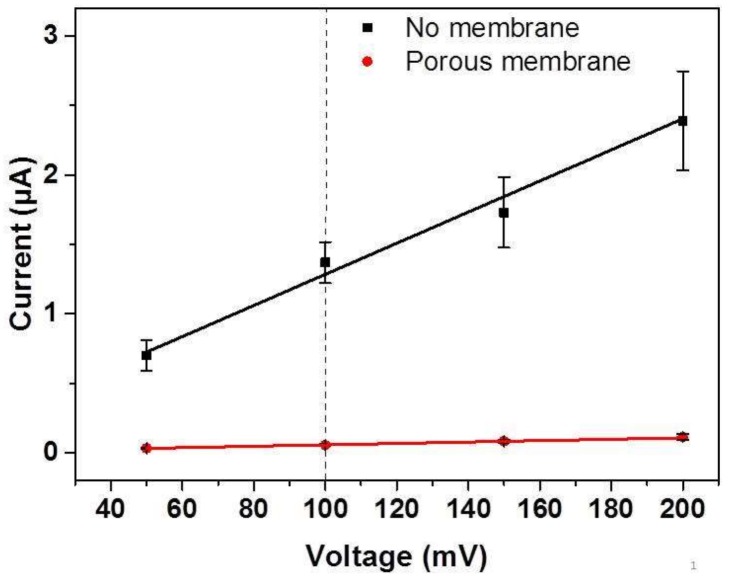
Current/voltage characteristics of an aqueous SPP buffer solution for the different setups described in the text. The dashed line marks the voltage used for the electrophoresis-driven disc insertion at 100 mV. Besides the mean values of the currents, the standard deviations and the linear regressions are shown in the graph.

Before we compared the insertion results of both of our insertion approaches, we had to prove by a detailed electrochemical current-voltage (I/U) characterization of the prepared membrane whether the SSM was applicable for electrophoretic insertion experiments. For that purpose, an aqueous 75 mM sodium potassium phosphate (SPP) buffer solution was used as an electrolyte in this study, and three different setups were investigated (Figure S1a–c). The two buffer compartments were separated: (1) by a frame where the membrane was removed by reactive ion etching (RIE) and just the pyramid window remained (i.e., no blocking of ion diffusion, Figure S1a); (2) by a porous membrane with conical pores (i.e., partial blocking of the ion diffusion, Figure S1b); or (3) by an SN substrate without pores (i.e., complete blocking of ion diffusion, Figure S1c). The I/U characteristics in Figure 5 exhibit an ohmic behavior for setups (1) and (2) in the range of 50–200 mV. Due to the limited sensitivity of the amperemeter and concomitant noise, voltages below 50 mV were not taken into consideration.

As the multimeter from Keithley used here has a resolution of 10 nA, which is too small to measure absolute values of very small currents (e.g., in the fA regime), the resistance of the setup shown in Figure S1c was determined by evaluating the slope of the regression of the measured currents and the applied voltages between −200 mV and +200 mV for an aqueous 100 mM KCl solution [31]. As a result, from the evaluation of the slope, a resistance of 11.7 GΩ was determined when a substrate without pores (i.e., complete blocking of ion diffusion) was used to separate the two compartments of the electrophoretic setup, showing that the leakage current between 50 mV and 200 mV is negligible. As mentioned in the experimental part, the electrophoresis-driven insertion of the nanodiscs was carried out at a potential of +100 mV (see the dashed line in Figure 5). For this potential, the measured currents were within the range that can be determined reliably by the multimeter applied.

Figure 5 shows that at each potential tested, the overall resistance of the porous membrane (red symbols) was significantly lower compared to the resistance of the setup without the membrane (black symbols), in agreement with the reduced cross-section area available for charge carrier exchange in the case of the porous membrane. Nevertheless, this result demonstrates the existence of a potential drop along the longitudinal axis of the pores, which can be applied for electrophoresis-driven insertion of the charged nanodiscs.

### 2.3. Direct Observation of TMV Nanodisc Incorporation by SEM

The incorporation of macromolecules, TLNPs or protein pores into solid state membrane pores is usually affected by the Brownian motion and concentration gradients of the macromolecules along the membrane cross-section, as well as by the interaction between the bio-organic inlays and the inorganic inner surface of the pores. To compare the efficiency of both above mentioned insertion strategies for the nanodiscs into the SSM pores, i.e., the concentration gradient- and the electrophoresis-driven insertion, scanning electron microscopy images of the membranes before and after the corresponding insertion experiment were evaluated.

**Figure 6:**
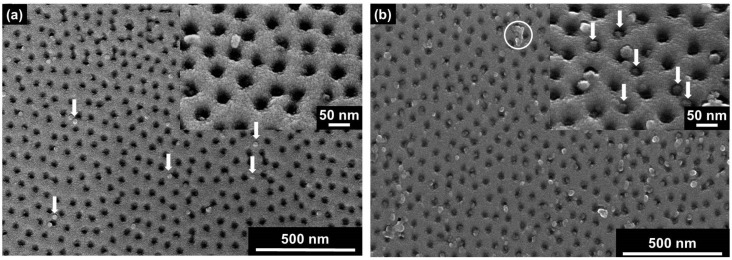
Concentration gradient-driven versus electrophoresis-driven insertion of TMV nanodiscs into SSM pores. (**a**) SEM image of a membrane after the 24-h diffusion experiment; (**b**) the membrane two hours after the electrophoresis experiment using a potential of +100 mV.

Upon the concentration gradient-driven approach, one compartment of the experimental setup was filled with a buffered TMV nanodisc suspension in contact with the top side of the SSM, while a second compartment contacting the back side of the membrane was filled with buffer solution without nanodiscs (Figure S2a). The nanodiscs moved along the gradient into the pores of the SSM and were trapped inside. Figure 6a shows an SEM image of a representative area of an SSM, which was placed in between both compartments of the liquid cell for 24 h. Several TLNPs can be identified on the SSM image (represented by white arrows). However, most of these soft-matter particles are placed on the surface and not inside the pores.

As only a few nanodiscs were inserted by the concentration gradient-based diffusion approach, it was tested if the insertion rate could be increased by way of the alternate electrophoresis-driven strategy. For this purpose, the experimental setup for the concentration gradient-driven insertion was changed by mounting one electrode in each compartment of the setup in a way that the electrode contacting the nanodisc dispersion was used as the cathode (Figure S2b). A potential of 100 mV was applied across the electrophoretic cell to direct the nanodiscs into the SSM pores. Figure 6b shows a representative image of an SSM section after electrophoresis-driven particle insertion into the membrane pores. Compared to Figure 6a, more nanodiscs can be detected on the surface of the membrane, and many of the pores are filled with TLNPs (represented by white arrows in the inset of Figure 6b). Furthermore, some nanodiscs have formed larger aggregates, which can be identified due to their increased dimensions (an example is marked with the white circle in Figure 6b) and which do not appear after applying solutions devoid of nanodiscs. In summary, the electric-field-based method was proven to direct more TLNPs into the SSM pores whilst taking less time compared to the concentration gradient-driven experiments. 

To get more quantitative information about how much is the percentage of the pores occupied by nanodiscs, the overall cross-sectional area of pores can be estimated by image analysis. ImageJ software was used to distinguish between pore areas and the surface of the membrane by evaluating the dark/bright contrast. The porosity (*P*) can be defined as:

$$P=\;\frac{\mathrm{Total}\;\mathrm{cross}\;\sec\mathrm{tion}\;\mathrm{area}\;\mathrm{of}\;\mathrm{pores}}{\mathrm{Total}\;\mathrm{area}\;\mathrm{of}\;\mathrm{image}}\times 100$$

The porosity of a fresh membrane (Figure 3a) was compared with those of membranes that were used for concentration gradient-driven (Figure 6a) and electrophoresis-driven (Figure 6b) experiments. The fresh membrane with 100% unoccupied pores exhibited 8% porosity (Figure S3a). After concentration-gradient-driven insertion experiments, the porosity remained almost constant at about 8%, because most of the discs were attached to the surface of the membrane, leaving most pores unoccupied (Figure S3b). In the case of electrophoresis-driven insertion, the porosity decreased significantly to about 5% (Figure S3c), indicating a more effective disc insertion than in the concentration gradient-driven case. In other words, the porosity of the SSM after electrophoretic disc insertion was reduced to 63% of the porosity of the freshly-prepared membrane, which means that 63% of the membrane pores remain unoccupied, while 37% of the pores are occupied by nanodiscs.

**Figure 7:**
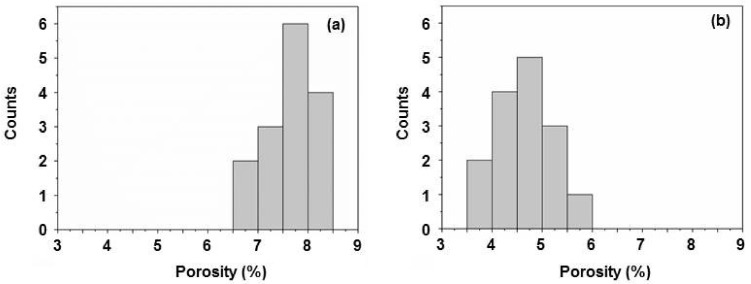
Distribution of the porosity of the SSM (**a**) after concentration gradient-driven nanodisc insertion (evaluation for four different sample areas) and (**b**) after electrophoresis-based insertion (evaluated for five different sample areas).

Alternative quantitative information on how much is the percentage of the SSM pores occupied by nanodiscs can be obtained by the evaluation of the SEM images of the membranes by counting those pores occupied by discs on several randomly-chosen membrane areas. By definition, the occupied pores do not contribute to the calculated porosity any longer. The results are shown in Figure 7. The porosities in the case of either concentration gradient-driven insertion (Figure 7a) or electrophoresis-based disc insertion (Figure 7b) into the SSM pores were between 6.5% and 8.5%, and between 3.5% and 6%, respectively. The resulting porosities are comparable to those determined by the ImageJ software-aided evaluation described above.

To verify the results obtained by the SEM data evaluation, we used a setup according to Figure S2b and determined the resistance before and after disc insertion. This revealed an increase of the resistance from 2.7 to 4.8 MΩ, respectively, for a potential of 100 mV. This increase can be qualitatively explained by the reduction of the permeable pore area due to the incorporation of nanodiscs into the SSM pores. To estimate to what extent this increase in SSM resistance correlated with the SEM-based results on the ratio between SSM pores without and with nanodiscs after electrophoresis-driven nanodisc insertion, a more detailed inspection is necessary. As shown in the Supplementary Information (Section 4), the total resistance of the porous SSM can be calculated by the help of the assumption that the pores harbor individual, but identical resistances, which are connected in the form of a parallel circuit (see Figure S4). As the total number of pores of the 20 µm × 20 µm SN membrane is 77,200, the resistance of one pore can be deduced, when the total resistance of the membrane is known. In the case that a buffer without nanodiscs was used as the electrolyte, a total resistance of 2.7 MΩ was measured, resulting in a calculated resistance of about 208 GΩ for an individual pore. In the case of a nanodisc inserted in an SSM pore and with the assumption of a significant decrease in that pore’s conductivity contribution down to a negligible amount, the increase in total resistance from 2.7 MΩ before nanodisc application to 4.8 MΩ thereafter can be assigned to the reduced number of unoccupied SSM pores. With the known total hybrid membrane resistance of 4.8 MΩ and the resistance of an unoccupied SSM pore of around 208 GΩ, the calculated total number of unoccupied pores after applying a nanodisc suspension would arithmetically amount to 43,417. This means that 56% of the SSM pores remain unoccupied while 44% of the pores are occupied by nanodiscs. In conclusion, we have demonstrated that for both evaluation methods (either based on the SEM images or on the I/U characteristics), the ratio of occupied pores/unoccupied pores is roughly 60/40.

## 3. Materials and Methods

### 3.1. Preparation of the Solid State Membrane

For the preparation of the nanoporous membrane, commercially-available, free-standing silicon nitride (SN) substrates from Silson Ltd. (Southam, England) were used, with a size of 20 µm × 20 µm and a thickness of 50 nm, supported by a 5 mm × 10 mm Si support carrier frame with a pyramidal opening at the backside to provide access to the SN substrate. The preparation of the porous membrane was carried out according to the protocol described by Seidenstücker et al. [33]. The silicon nitride membrane was coated with 90 nm of silicon oxide using electron beam deposition. Then, an inverse micelle technique was applied by dipping the membrane into a suspension of poly(styrene)-block-poly(2-vinylpyridine) di-block-copolymer (Polymer Source Inc., Dorval, QC, Canada) micelles loaded with gold salt HAuCl_4_ (Sigma-Aldrich, Darmstadt, Germany) in toluene (Merck, Darmstadt, Germany). Hexagonal arrays of gold particles were obtained after exposing the samples to hydrogen plasma (Figure S5a) [31,41]. Due to the size limitation of the gold particles that can be achieved by this micelle deposition technique, a photochemical size enlargement process was carried out to increase the particle diameter size to around 20 nm in accordance with [42] (Figure S5b). In a further step, RIE (OXFORD PlasmaLab 80 Plus ICP65, Yatton, Bristol, England) with a gaseous mixture of CF_4_ and CHF_3_ (2:20 sccm, 10 mTorr, 25 °C) was used to remove the silicon oxide [43]. The gold particles work as masks during the etching process and cause a pillared structure to remain underneath each gold particle (Figure S5c). To convert pillars into pores, a mask inversion process was employed. A 15-nm chromium layer was thermally deposited onto the sample (Figure S5d). Subsequently, the pillared structure was removed by argon sputtering, which hits the surface under a small incidence angle of 6° (Figure S5e). Finally, the repetition of the RIE process creates open pores in the unmasked part of the surface (Figure S5f).

### 3.2. Viral Nanodisc Preparation

Nucleoprotein pore adapters were generated from tobacco mosaic virus (TMV)-based components, i.e., single-stranded ribonucleic acid (ssRNA) and coat protein (CP), as described in Altintoprak et al., 2017 [32]. This section briefly describes the fabrication procedure. A short ssRNA with a length of 204 nucleotides (to be further referred to as 204 nt RNA) containing the origin of assembly (OAs) of TMV was generated through in vitro transcription with a MEGAscript^®^ T7 High Yield Transcription Kit (Ambion, Austin, TX, USA). For in vitro transcription, 100 ng of DNA template containing cDNA of the TMV genome sequence position 5350–5531 (NC_001367.1, [44]) under the control of the T7 promoter in a pGEM-T-Easy vector (Promega, Mannheim, Germany) were used per 20-µL reaction. For a description of the cloning procedure and PCR amplification, see Altintoprak et al. [32]. The reaction was carried out for 6 h, and RNA products were precipitated by final concentrations (f.c.) of 4.5 M LiCl and 30 mM EDTA over night at −20 °C. The resulting pellet was dissolved in dimethyl dicarbonate-treated deionized water. Two variants of TMV CPs were used for the assembly: the TMV wild type (CP_wt_) [45] and a genetically modified CP mutant (CP_Lys_) with an amino acid residue exchange of a threonine (Thr, T) at position 158 to lysine (Lys, K) [46]. For CP preparation, TMV particles were isolated according to Gooding and Hebert [47] from systemically-infected *Nicotiana tabacum* ‘Samsun’ nn plants. RNA-free CPs were prepared according to the protocol of Fraenkel-Conrat [48] using acidic particle disassembly. CPs were suspended in 75 mM sodium phosphate (SPP) buffer at a pH of 7.2. In vitro assembly was carried out in a mixed assembly approach according to Eiben et al. [49]. A 6-µL amount of 10 mg/mL CP_Lys_ and 14 µL of 10 mg/mL CP_wt_ were combined with 76 µL of 75 mM SPP (pH 7.2) and incubated at 10 °C overnight to disassemble aggregates of discs and protohelices [50]. After disassembly, the CP_wt_-CP_Lys_ mixture was incubated for 48 h at room temperature to allow the formation of discs and protohelices [50] with a homogenous distribution of both CP variants in each disc or protohelix. The in vitro assembly was initialized by the addition of 4 µL of 3 µg/µL 204 nt RNA, i.e., an excess of RNA sufficient to convert all of the assembly-competent CP into nanodisc structures (thus yielding 100 µL of a nanodisc suspension with in total 200 µg CP). The mixture was then incubated overnight at 25 °C. For the electrophoresis-driven insertion into the SSM pores, the mixed-assembled TMV-like particles were diluted 1:10 in 75 mM SPP at a pH of 7.2, resulting in a CP (and thus a similar nanodisc) concentration of 0.2 mg/mL, corresponding to 1.04 × 10^14^ nanodiscs per mL.

### 3.3. Electrochemical Characterization of the Membrane and Incorporation of the Viral Nanodiscs

For the electrochemical characterization of the membrane and the incorporation of the TMV nanodiscs into the pores of the SSM membrane, the setups shown in Figures S1 and S2, respectively, were used. Both setups consist of two compartments of an electrophoretic cell consisting of poly(methyl methacrylate) (PMMA) with a volume of 300 µL each, which were separated: (i) by the silicon nitride substrate without pores; (ii) by the Si frame without membrane; or (iii) by the porous SSM. 

For the membrane characterization, both compartments of the setups shown in Figure S1a,b were filled with 300 µL of a 75 mM SPP buffer (pH 7.2) after a pre-wetting of the membrane was carried out by pure ethanol. The current/voltage (I/U) characteristics were investigated by electrophoretic experiments with a pure SPP buffer lacking discs, to serve as a conducting electrolyte. The currents were determined for 50 mV, 100 mV, 150 mV and 200 mV by measuring the current values five times and calculating the mean current values. The electric field was generated by two Ag/AgCl_2_ electrodes inserted into the solutions of the compartments. With the direct current (DC) power supply GPD-X303S (GW Instek Co., New Taipei City, Taiwan), a potential of 100 mV between both electrodes was applied for 2 h. Two LabVIEW controlled multimeters of the type Keithley 2000 (Tektronix, Germering, Germany) were connected to a computer to record both the current and the potential during the electrophoretic experiment. To prove that the leakage current of the electrophoretic cell is negligible, the resistance of the setup shown in Figure S1c with a non-porous SN substrate was determined by evaluating the slope of the regression of the measured currents and the applied voltages between −200 mV and +200 mV for an aqueous 100 mM KCl solution by using a patch clamp amplifier of the type EPC 08 from HEKA (Lambrecht, Germany).

For the disc insertion experiment, the compartment in contact with the side of the SSM with smaller pore apertures was filled with 300 µL of a 75 mM SPP buffer (pH 7.2). The other compartment was filled with 300 µL of a suspension of nanodiscs in the same buffer. For the implantation of the nanodiscs into the pores, two different techniques were used: (1) an insertion driven by the concentration gradient of the nanodiscs (Figure S2a), where after a diffusion time of 24 h, the diffusion was stopped; and (2) an electrophoresis-based insertion, where the nanodiscs move along an electric field inside the conical pores of the SSM. For the second approach, the same experimental conditions and setup as for the latter technique were used, but applying a potential of 100 mV for 2 h between both compartments, which was generated by two Ag/AgCl_2_ electrodes, where the electrode contacting the disc suspension is used as the cathode (Figure S2b). After each experiment, salt crystals formed on the SSM surface were removed by immersing the membranes in deionized water for 2 min. In all cases, each SSM membrane was pre-incubated with 20 µL of pure ethanol directly before use to enable an efficient wetting of the pore channels by the buffer solution.

To support the results obtained by electron microscopy and as a complementary proof for the nanodisc insertion into the SSM pores, the resistance of the setup was determined by measuring the current at 100 mV after 7000 s (i) with the pure SPP buffer solution without disks as the conducting electrolyte and (ii) with the same buffer solution with nanodiscs by using the same experimental equipment described above for the insertion experiment.

### 3.4. Electron Microscopy

A Hitachi S-5200 scanning electron microscope (SEM) (Hitachi, Tokyo, Japan) with a transmission mode option was used to investigate the membrane before and after the concentration gradient- or electrostatically-driven diffusion of the TMV nanodiscs. By using the secondary electron emission, a top view image of the SSM surface could be generated, while a detector placed in the backside of the sample recorded transmitted electrons. Using this method, it was possible to simultaneously record surface images of the SSM and the size of the smaller opening of the conical pores through transmitted electrons. It was found that TMV nanodiscs on the membrane were detectable exclusively after a 2 nm-thick platinum layer was deposited on the substrate. This is due to (1) the low yield of secondary electrons in the case of the CPs forming the TMV nanodiscs and (2) a significant charging effect by the electrons during SEM investigation. Alternatively, TMV nanodiscs deposited on a carbon/Formvar^®^-covered copper grid can be visualized under a transmission electron microscope (TEM) after negative staining with 2% uranyl acetate according to Altintoprak et al. [32]. A Tecnai G2 Sphera TEM (FEI, Hillsboro, OR, USA), operating at an acceleration potential of 120 kV and equipped with a 16-megapixel camera (TemCam F416, TVipS, Gauting, Germany) was used for the investigation of the nanodiscs shown in Figure 3c.

### 3.5. Evaluation of SEM Images

To determine how much the areas of the pore apertures contribute to the overall membrane area (denominated as porosity in the following), analysis with the ImageJ software [36] was performed as follows: First, the SEM images were converted to greyscale. Then, the brightness threshold value in each image was set to create black/white images, so that the pore areas remained black and other surrounding areas or filled pores changed to white. By using the “Analyze Particle” option in ImageJ, each black area (= unfilled pore) can be detected individually, and the edges of the black areas are marked with a black frame. Figure S3 shows the results of this evaluation where the pore areas that are used for porosity calculation are marked with the black frame. In both cases—the electrophoresis-driven and the concentration gradient-driven insertion—15 different areas of each sample were evaluated this way.

## 4. Conclusions

The described approach to create biohybrid membranes with exactly defined protein-lined pores is motivated by the knowledge that the preparation of large-area porous membranes using “self-controlling” strategies (i.e., anodic oxidation of aluminum or gold-particle-based lithography technique used in this work) usually results in a broad distribution of pore diameters or irregular pore shapes. The preparation of pores with both an exact diameter and regular shape can be typically realized by top-down techniques (e.g., focused ion beam or electron beam techniques) for a single or a few pores only. Although those approaches are helpful in particular for basic research, they are not practical for the high-throughput preparation of multiple pore membranes or of membranes with a multitude of identical nanopores. Therefore, the insertion of preassembled, perfectly-defined biological disc-shaped building units in a large area of porous SSMs through a “self-controlling” approach might be a promising way towards a reproducible mass production of hybrid membranes. This method can create exactly defined pores and might overcome several drawbacks of large-area porous membranes produced so far after further optimization. It has to be mentioned that the nanodiscs’ insertion principle that is described in this work does not take into account how the nanodiscs are trapped and positioned in the SSM pores and that there will certainly remain gaps in between the outer edges of the nanodiscs and the inner walls of the SSM pores. Solving this issue will definitely constitute a big challenge in the future. However, although not shown here, a strategy for sealing the gaps by site-selective bio-inspired mineralization, which can be initiated by a corresponding chemical functionalization and composition of the coat protein assembly, is under development in order to overcome this challenge. Particularly, the latter aspect shows that the CP subunits of the nanodiscs play a crucial role in optimizing the nanodiscs’ properties. As a further option, the central protein pore diameters of the plant virus-based nanodiscs may be controlled by integrating different variants of TMV CP subunits, which have correspondingly altered steric dimensions. Additionally, the chemical character of the pores might be modulated by genetic engineering and/or chemical functionalization of the CPs used for nanodisc assembly. This gives maximum flexibility in designing nanopores with well-defined properties, tailored to the intended application. It has to be mentioned here that the preparation of nanoporous SSMs by colloidal approaches based on the self-assembly of micelles can be used for large area applications. However, with such membranes, it is not possible to investigate individual pores, e.g., in terms of their permeance for certain ions or molecules. To this end, top-down procedures exploiting, e.g., electron or ion beam techniques have to be chosen, by which individual pores with defined diameter, shape and inter-porous distances can be prepared and investigated in a pre-defined manner. The combination of SSMs prepared by top-down techniques and TMV-derived nanodisc inlays may, therefore, be a future option for highly analyte-specific detection layouts.

## Supplementary Materials

The following are available online at http://www.mdpi.com/2079-4991/8/4/237/s1. Figure S1: Experimental setup for I/U characteristics of the membrane, Figure S2: Experimental setup for the nanodisc insertion experiment, Figure S3: Images created by ImageJ software, Figure S4: Detailed considerations on the resistances of unoccupied SSM pores and pores occupied by nanodiscs, Figure S5: SEM images of the intermediate states during the preparation of the solid state membrane.
