# Supplementary material for: Electrochemically-Driven Insertion of Biological Nanodiscs into Solid State Membrane Pores as a Basis for “Pore-In-Pore” Membranes
Source: Nanomaterials (Basel). 2018 Apr 13;8(4):237. doi: 10.3390/nano8040237 (PMC5923567; doi:10.3390/nano8040237)
Supplement: Supplementary file 1 [file nanomaterials-08-00237-s001.pdf]

Electrochemically-Driven Insertion of Biological Nanodiscs into Solid State Membrane Pores as a Basis for "Pore-in-Pore" Membranes

Farid Farajollahi[1,#], Axel Seidenstücker[2,#], Klara Altintoprak[3], Paul Walther[4], Paul Ziemann[2], Alfred Plettl[2], Othmar Marti[5], Christina Wege[3] and Hartmut Gliemann[1,*]

[1] Institute of Functional Interfaces (IFG), Karlsruhe Institute of Technology (KIT), 76344 Eggenstein-Leopoldshafen, Germany; farid.farajollahi@kit.edu; hartmut.gliemann@kit.edu

[2] Institute of Solid State Physics, University of Ulm, 89081 Ulm, Germany; dr.a_seid@gmx.de; paul.ziemann@uni-ulm.de; alfred.plettl@gmail.com

[3] Institute of Biomaterials and Biomolecular Systems, University of Stuttgart, 70569 Stuttgart, Germany; klara.altintoprak@bio.uni-stuttgart.de; christina.wege@bio.uni-stuttgart.de

[4] Central Facility for Electron Microscopy, University of Ulm, 89081 Ulm, Germany; paul.walther@uni-ulm.de

[5] Institute of Experimental Physics, University of Ulm, 89081 Ulm, Germany; othmar.marti@uni-ulm.de

[#] Both authors contributed equally
* Correspondence: hartmut.gliemann@kit.edu; Tel.: +49 721 608 26435

Supplementary Material

1. Experimental setup for I/U characteristics of the membrane

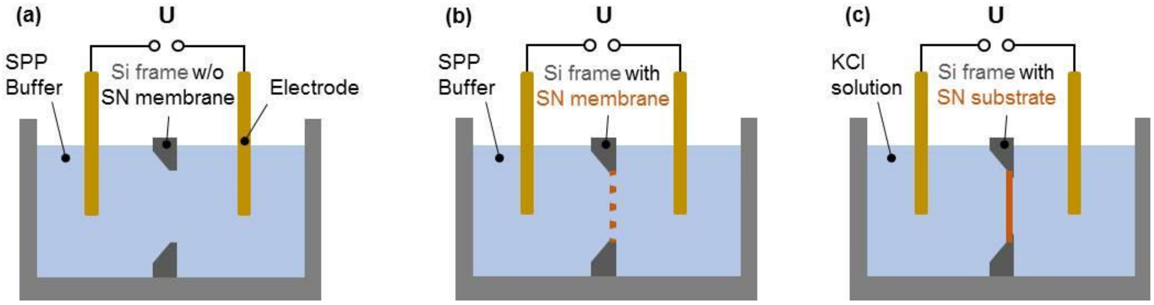

Figure S1. Scheme of the two-compartment cell used for the electrophoresis experiments. With these three setups, the experimental data for Figure 5 were obtained. (**a**) Open membrane window. (**b**) SSM with conical pores. (**c**) An SSM without pores.

2. Experimental setup for the nanodisc insertion experiment

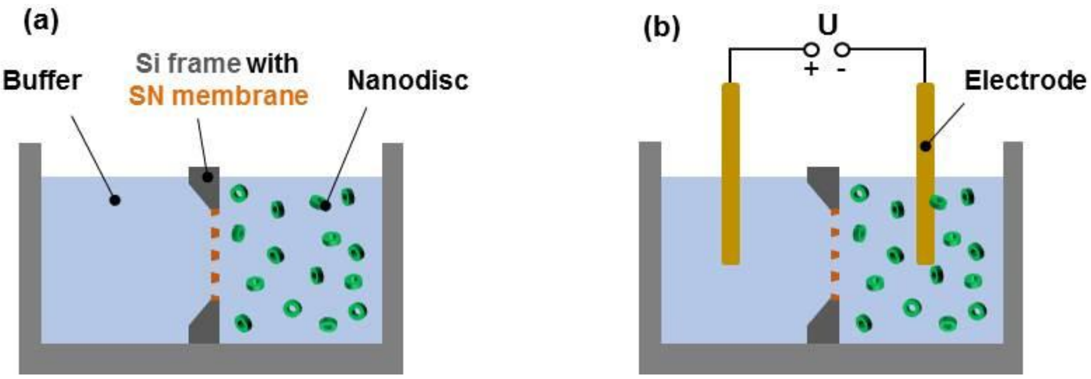

Figure S2. Scheme of the two-compartment cells used for the nanodisc insertion experiments. (**a**) The concentration-gradient-driven insertion. (**b**) The electrophoresis-driven insertion.

3. Images created by ImageJ software

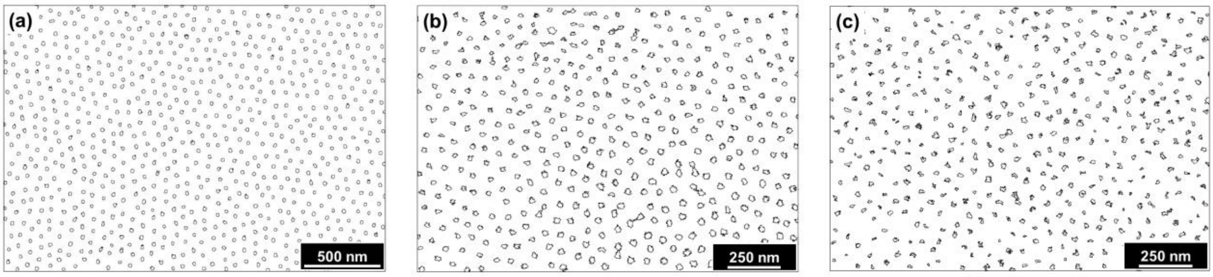

Figure S3. Software-created images for pore area detection corresponding to images (**a**) Figure 3a, (**b**) Figure 6a, and (**c**) Figure 6b.

4. Detailed considerations on the resistances of unoccupied SSM pores and pores occupied by nanodiscs

To calculate the average total number of nanopores in the SSM, the pores were counted for 1 μm^2 (see Figure S4a). As the total area of the SSM is given with 20 µm x 20 µm and the number of pores per μm^2 is 193, the total number of pores for a typical SN membrane of the present work is 400 x 193 = 77,200.

As the SSM pores can be seen as a parallel circuit of individual resistances ($R_{pore\ i}$, see Figure S4b) and assumed that the resistance of each pore is identical, the total resistance of the SSM can be calculated by the following equation:

$$\frac{1}{R_{total}} = \frac{1}{R_1} + \frac{1}{R_2} + \frac{1}{R_3} + \frac{1}{R_4} + \cdots = \frac{N_{non-occupied}}{R_{pore}}$$

As the measured total resistance of the membrane in the case of the pure electrolyte (i.e. without nanodiscs) is 2.7 MΩ and $N_{non\text{-}occupied}$ is 77,200 (see above), R_{pore} for one SSM pore can be calculated to 208.4 GΩ according to the equation:

$$R_{pore} = N_{non-occupied} \cdot R_{total}$$

Assumed that the contribution to the membrane's overall conductivity of an SSM pore occupied by a nanodisc can be neglected, the increased resistance of 4.8 MΩ of the SSM measured after the electrophoresis-driven nanodisc insertion is due to a reduced number of non-occupied SSM pores compared to the case described before which can be calculated to 43,417 according to the equation:

$$N_{non-occupied} = \frac{R_{pore}}{R_{total}}$$

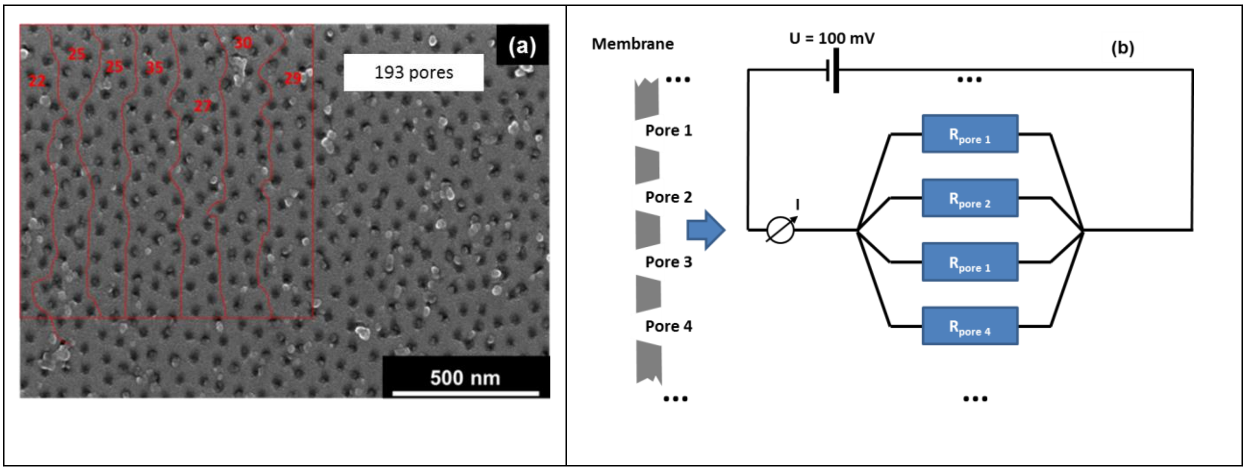

Figure S4. (a) SEM image of a representative area of an SSM used for the calculation of the average amount of total SSM pores. The red numbers represent the amount of counted pores within the areas separated by the red lines. (**b**) shows the equivalent circuit for the SSM pores used for the calculations.

5. SEM images of the intermediate states during the preparation of the solid state membrane

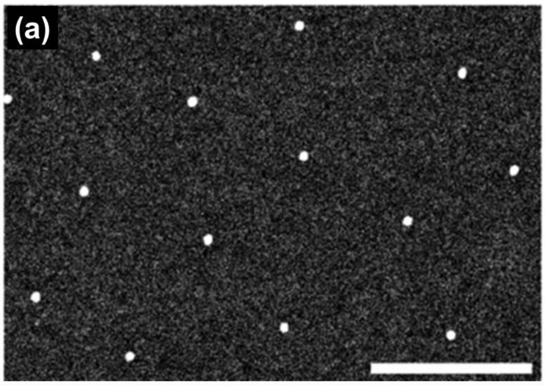

(a)

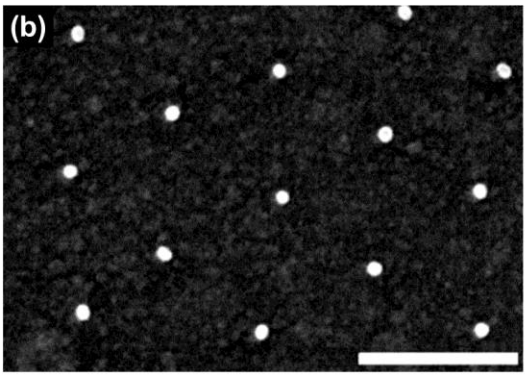

(b)

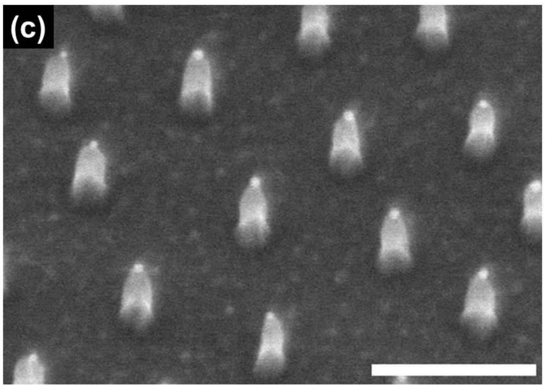

(c)

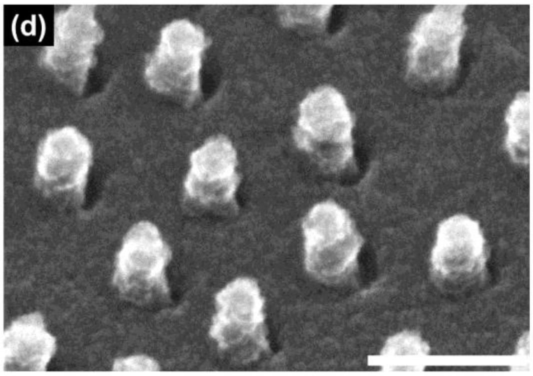

(d)

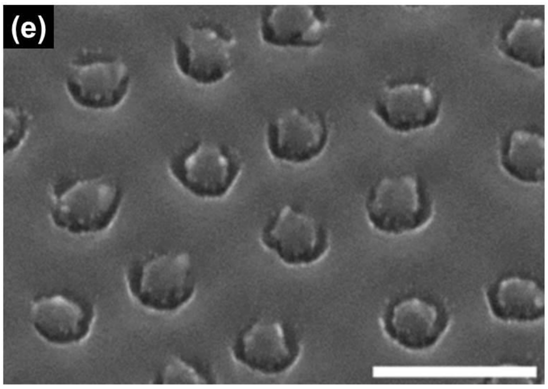

(e)

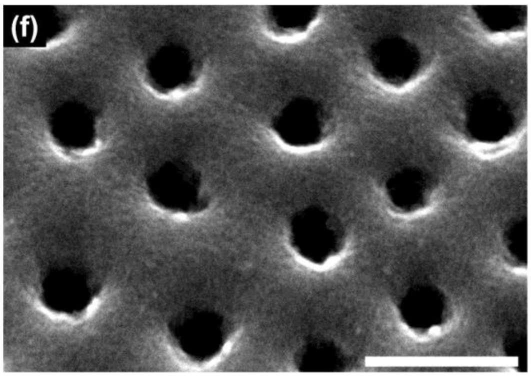

(f)

Figure S5. SEM images of the intermediate states during the preparation of the solid state membrane. (**a**) A hexagonal array of gold particles after exposing the samples to hydrogen plasma [31], (**b**) after the photochemical size enlargement process, (**c**) after the etching process, (**d**) after the deposition of a 15 nm chromium layer, (**e**) after argon sputtering, and **(f)** after pores are opened by reactive ion etching (scale bar: 100 nm).
